# Chances and challenges of photon-counting CT in musculoskeletal imaging

**DOI:** 10.1007/s00256-024-04622-6

**Published:** 2024-03-05

**Authors:** Charbel Mourad, Lucia Gallego Manzano, Anaïs Viry, Ronald Booij, Edwin H. G. Oei, Fabio Becce, Patrick Omoumi

**Affiliations:** 1 Department of Radiology, Lausanne University Hospital and University of Lausanne, Lausanne, Switzerland; 2 Department of Diagnostic Imaging and Interventional Therapeutics, Hôpital Libanais Geitaoui-CHU, Beyrouth, Lebanon; 3 Institute of Radiation Physics (IRA), Lausanne University Hospital (CHUV) and University of Lausanne (UNIL), Lausanne, Switzerland; 4 Department of Radiology & Nuclear Medicine, Erasmus Medical Center, Rotterdam, The Netherlands; 5 Center for Medical Image Science and Visualization (CMIV), Linköping University, Linköping, Sweden

**Keywords:** Photon-counting CT, Musculoskeletal imaging, Spectral imaging, Crystal arthropathies, High-resolution imaging

## Abstract

In musculoskeletal imaging, CT is used in a wide range of indications, either alone or in a synergistic approach with MRI. While MRI is the preferred modality for the assessment of soft tissues and bone marrow, CT excels in the imaging of high-contrast structures, such as mineralized tissue. Additionally, the introduction of dual-energy CT in clinical practice two decades ago opened the door for spectral imaging applications. Recently, the advent of photon-counting detectors (PCDs) has further advanced the potential of CT, at least in theory. Compared to conventional energy-integrating detectors (EIDs), PCDs provide superior spatial resolution, reduced noise, and intrinsic spectral imaging capabilities. This review briefly describes the technical advantages of PCDs. For each technical feature, the corresponding applications in musculoskeletal imaging will be discussed, including high-spatial resolution imaging for the assessment of bone and crystal deposits, low-dose applications such as whole-body CT, as well as spectral imaging applications including the characterization of crystal deposits and imaging of metal hardware. Finally, we will highlight the potential of PCD-CT in emerging applications, underscoring the need for further preclinical and clinical validation to unleash its full clinical potential.

## Introduction

In the clinical practice of musculoskeletal imaging, CT is used for a wide range of indications, either as a standalone modality or as a complement to MRI. While the latter remains the diagnostic modality of choice for analyzing bone marrow and low-contrast structures such as soft tissues, CT is preferred for the assessment of high-contrast structures, in particular mineralized tissues, including bone and crystal deposits [1]. Advanced imaging techniques such as CT arthrography may also be used for the assessment of the internal derangement of joints whenever MRI is unavailable or contraindicated [2, 3]. Additionally, imaging patients with metallic hardware is typically less challenging using CT compared to MRI. CT further offers additional benefits compared to MRI, such as superior spatial resolution and a less complex implementation of dynamic imaging [4].

The development in CT technology has been closely related to advances in computer technology (e.g., higher processing speed and capacity allowing for advanced reconstruction algorithms, including AI-based algorithms), X-ray production (e.g., cone-beam CT, synchrotron imaging), and X-ray detection (e.g., newer generations of detectors). In particular, advances in detector technology and computer processing have enabled the advent of dual-energy CT (DECT) and its use in clinical practice, paving the way to the development of new applications in musculoskeletal imaging, including material characterization and improved methods for metal artifact reduction.

More recently, a newer generation of detectors, known as the energy-resolving photon counting detectors (PCDs), has emerged and the first FDA approval for a clinical scanner utilizing these detectors was received in 2021 [5, 6]. The major difference between PCDs and conventional energy-integrating detectors (EIDs) is their capacity to register and resolve the energy of each incoming X-ray photon. The technical features of these detectors confer multiple benefits to PCD-CT over EID-CT, such as superior spatial resolution, reduced noise, enhanced dose efficiency, and intrinsic spectral imaging capabilities. Therefore, PCD-CT presents theoretical advantages over EID-CT for virtually all CT applications in clinical practice. These advances have led some experts to consider the advent of PCD-CT as a revolutionary development in CT imaging [7, 8]. After a brief overview of the technical background, this review will describe how PCD-CT may influence existing applications of CT in musculoskeletal imaging, as well as emerging applications that could transition from the research domain into clinical practice. The technical challenges and current limitations in applying PCD-CT in clinical practice will also be highlighted.

### Technical background

#### Advantages of photon-counting detectors over conventional energy-integrating detectors

**Fig. 1:**
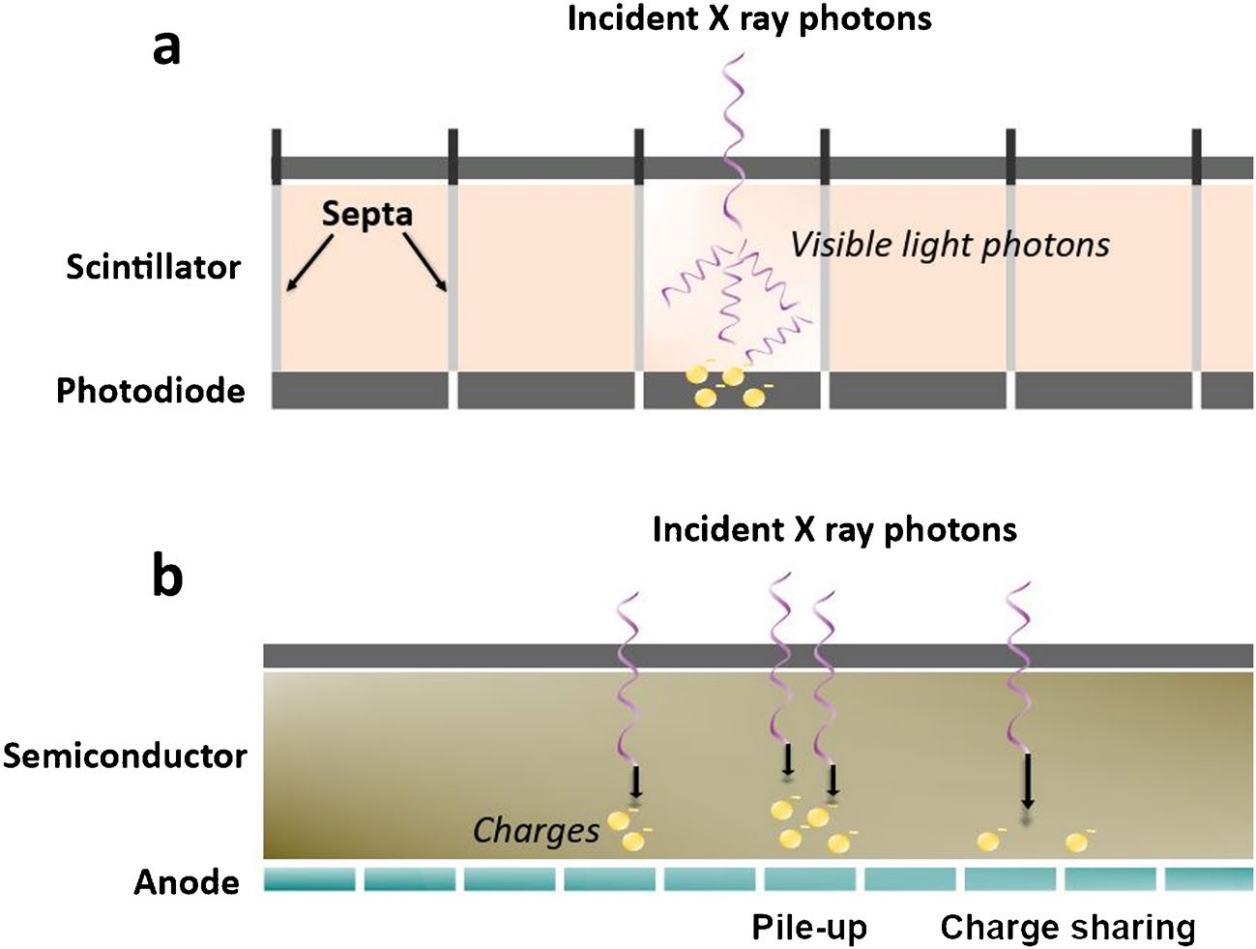
Energy-integrating detector (EID) vs. photon counting detector (PCD): **a** EID measures the total energy deposited by incoming X-rays. A scintillator transforms X-ray photons to visible light that is detected by a photodiode. The use of septa creates dead space; **b** PCD counts individual photons by directly converting photon energy to electric signal, offering energy discrimination for improved image quality and dose efficiency. Pile-up effect occurs when multiple X-ray photons arrive at the detector within a short time, causing overlapping signals and compromising accurate energy measurement. Charge sharing effect arises when the charge generated by a single X-ray photon is distributed between neighboring detector elements, leading to reduced spatial and energy resolution

Energy-resolving PCDs offer significant advantages over conventional EIDs, which are used in both conventional and dual-energy CT [9–11]. The key difference between EIDs and PCDs lies in how they detect X-ray photons. EIDs utilize scintillators to convert X-ray energy into visible light, which is then transformed into electrical signals using photodiodes (Fig. 1). EIDs sum the total energy deposited by X-ray photons over a certain range, combining the information without differentiating individual energy levels. This approach results in spectral information loss, limiting their ability to differentiate between materials with similar attenuation characteristics. In contrast, PCDs are made of semiconductors, which directly convert X-ray photons into electrical signals. This enables PCDs to register each incoming photon individually and classify them based on their energy levels, allowing for precise material discrimination and multi-energy imaging, while improving spatial resolution, and decreasing noise and/or radiation dose [5, 12–15].

Current PCD-CT systems exhibit variations in detector technology, energy resolution, and overall performance, leading to distinctions in their clinical applicability [16]. While the technology is still evolving, a series of prototype PCD-CT systems are currently available for both pre-clinical and clinical purposes. All major CT vendors have shown interest in PCD-technology, and at the time of writing (December 2023), Siemens Healthineers offers a commercially available system [8, 17]. Common to all manufacturers, PCD-CTs offer several notable advantages over conventional CT systems, including increased spatial resolution, improved dose efficiency, decreased noise, and increased spectral resolution [15, 17].

##### Spatial resolution

**Fig. 2:**
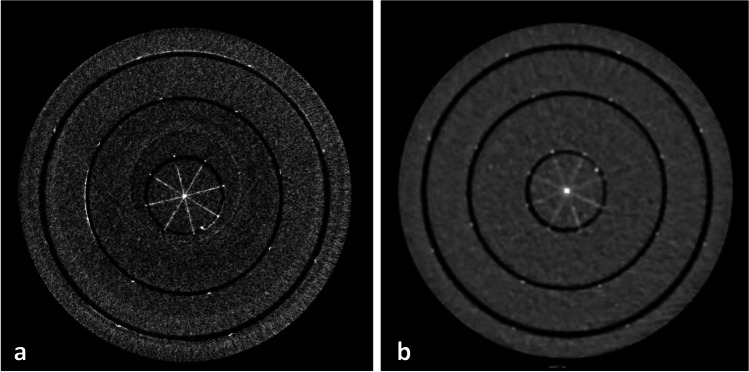
Improved spatial resolution between PCD-CT (**a**) and EID-DECT (**b**). Visual assessment of improved spatial resolution with a dedicated PCCT for extremities (**a**) compared to an EID-based DECT (**b**), despite lower dose at PCD-CT (3 mGy vs. 7 mGy with EID-DECT). Information about the phantom can be found elsewhere [https://doi.org/10.1002/mp.16313]

**Fig. 3:**
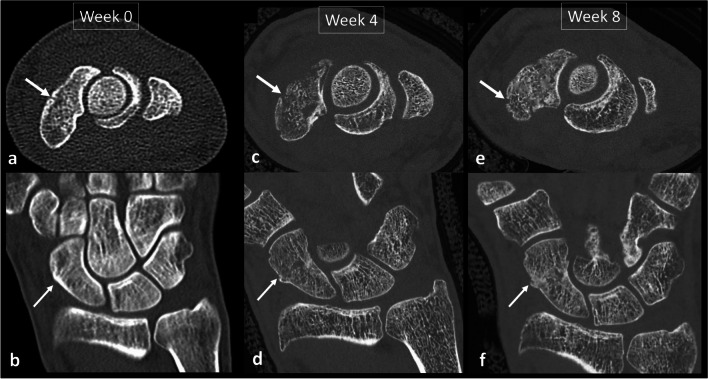
Improved spatial resolution between an EID-DECT (**a**, **b**) and clinical PCD-CT (**c**–**f**). A 47-year-old male with suspected scaphoid fracture (white arrows). The fracture was initially missed but retrospectively visible with low dose EID-CT of the wrist (CTDIvol_32_ 1.16 mGy) (**a**, **b**). Four weeks after suspected fracture (**c**, **d**), a PCD-CT was performed and demonstrated the fracture and the osteolytic changes as part of the repair process (CTDIvol_32_ 4.28 mGy). Note the higher resolution of PCD-CT with finer depiction of trabecular bone compared to DECT. PCD-CT 8 weeks after fracture (**e**, **f**) demonstrated a partly healed/immature bridging (CTDIvol_32_ 4.49 mGy)

The achievable spatial resolution in current CT systems is limited on the one hand by the size of the detector elements, and on the other hand by the use of septa, which create dead spaces (Fig. 1). PCDs are typically designed with pixel sizes significantly smaller than those commonly found in EIDs, resulting in substantially higher spatial resolution. Current PCD-CT systems propose pixel sizes at the isocenter ranging from 0.1 × 0.1 to 0.4 × 0.4 mm^2^. The direct conversion of photon energy to an electric signal without the use of reflecting septa not only improves spatial resolution compared to EIDs but also enhances geometric efficiency and radiation dose effectiveness, offering high-resolution images without the need for radiation-blocking grids or combs used in traditional EIDs, thereby delivering superior image quality and dose efficiency [5, 18]. The improved spatial resolution between a dedicated PCD-CT for extremity imaging and an EID-DECT is shown in Figs. 2 and 3.

##### Decreased noise reduction and radiation exposure

PCDs feature intrinsic higher dose efficiency compared to EIDs. This is due to several factors including reduced image noise and advanced energy-resolving capabilities. Indeed, PCDs can register and count each individual incoming X-ray photon. This advanced capability enables the precise quantification of the X-ray signal, effectively reducing the influence of electronic noise found in conventional EIDs. Through the implementation of specific energy thresholds, PCDs can effectively filter out lower-energy noise signals, leading to a significant reduction in overall noise and contributing to improved image quality, especially at lower radiation doses. As seen in the following sections, the higher dose efficiency of PCDs may be used to lower radiation dose for many applications of PCD-CT.

##### Spectral imaging

Spectral CT is an advanced medical imaging technique that enhances diagnostic capabilities of CT by exploiting the polychromatic information of X-rays. Around two decades ago, technological advances have allowed the clinical use of spectral imaging in the form of DECT, which combines two different X-ray energy spectra to differentiate materials, offering improved soft tissue contrast and reduced artifacts compared to conventional CT [19–24]. Since then, a broad range of DECT applications have become available [25–27]. By utilizing more advanced energy-resolving detectors compared to DECT, PCD-CT has the potential to take spectral imaging a step further [5, 12–15].

**Fig. 4:**
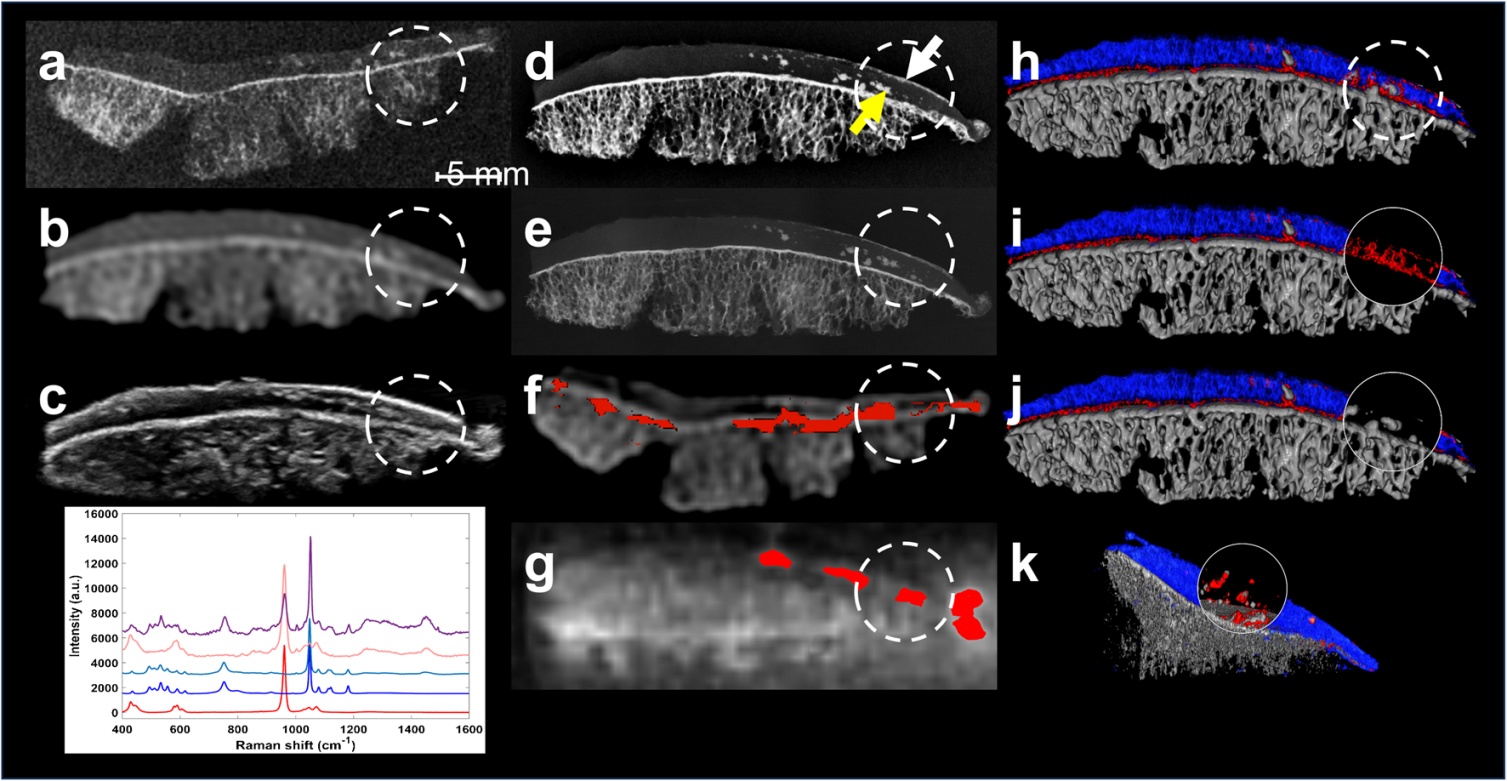
Detection and characterization of crystal deposits. **a** Digital radiography, **b** conventional CT, **c** ultrasound, **d** digital mammography, **e** micro-CT, **f** dual-energy CT (DECT), **g** ultrashort echo time (UTE) MRI, **h**–**k** multi-energy photon-counting-detector CT (PCD-CT). In UTE MRI, the articular cartilage calcium crystal deposition as a whole is color-coded in red due to its inability to discriminate between CPP and HA crystal deposits; in PCD-CT, the articular cartilage water content is color-coded in blue. While all conventional and advanced clinical imaging techniques were able to detect calcium crystal deposition within the articular cartilage (yellow arrow, dashed circle)—with varying levels of accuracy and sharpness/blur mainly due to spatial resolution differences—DECT and UTE MRI both failed to identify crystal deposits on the cartilage surface (white arrow, dashed circle). Additionally, although DECT, UTE MRI, and PCD-CT were all able to quantify calcium crystal deposition with varying accuracy, PCD-CT was the sole technique capable of distinguishing between CPP (color-coded in red) and HA (color-coded in white) crystal aggregates. (l) Raman spectroscopy was used as the reference standard (synthetic CPP and HA spectra are color-coded in blue and red, respectively) and confirmed the co- existence of CPP and HA crystals in two of the three cartilage scrapings/biopsies from the tibial plateau sample. Reprinted with permission from Bernabei et al. Multi-energy photon-counting computed tomography versus other clinical imaging techniques for the identification of articular calcium crystal deposition. Rheumatology (Oxford). 2021;60:2483–2485

**Fig. 5:**
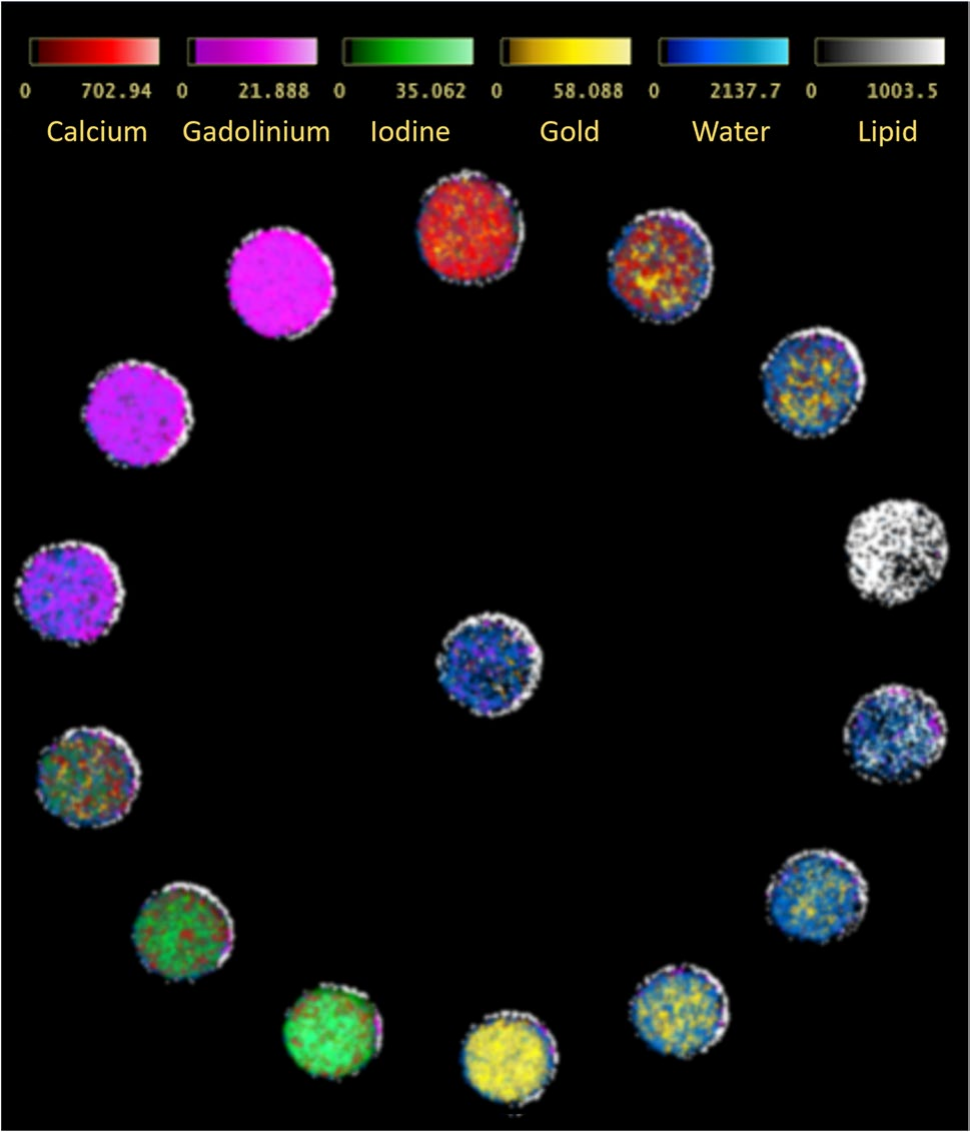
Material decomposition. Reconstructed image of a multi-contrast phantom obtained with a dedicated PCD-CT for extremities using a material decomposition algorithm. PCD-CT enables precise multi-material decomposition, distinguishing various components based on their unique energy-dependent attenuation properties; information about the phantom can be found elsewhere [https://doi.org/10.1002/mp.16313]

**Fig. 6:**
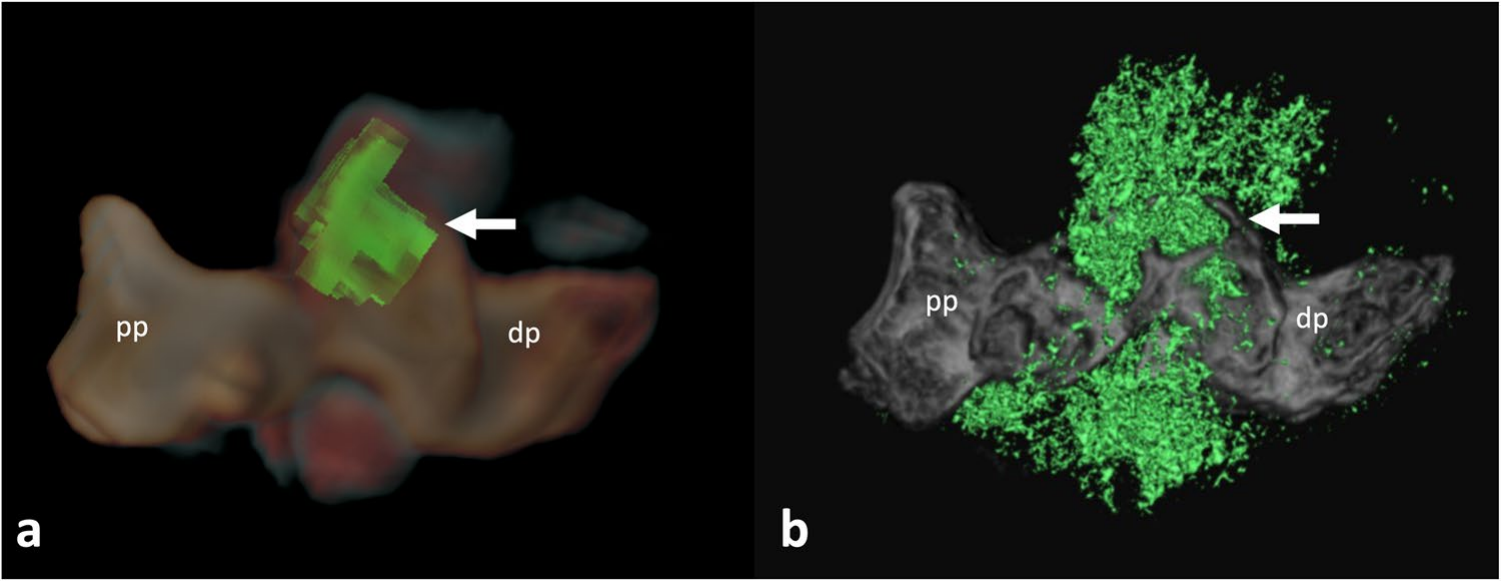
Crystal deposit detection and improved spatial resolution. EID-DECT (**a**) and PCD-CT (**b**) of a toe specimen obtained after amputation of first toe due to infection and gout. Volume rendering reconstructions with dorsal views (proximal phalanx (pp), distal phalanx (dp)), showing monosodium urate tophi (green). Note that on PCD-CT (**b**), the spatial resolution is higher, and the volume of crystal detected is larger. Also note the juxtaarticular erosion with typical overhanging edges (arrow). Specimen courtesy of Dr Sylvain Steinmetz

**Fig. 7:**
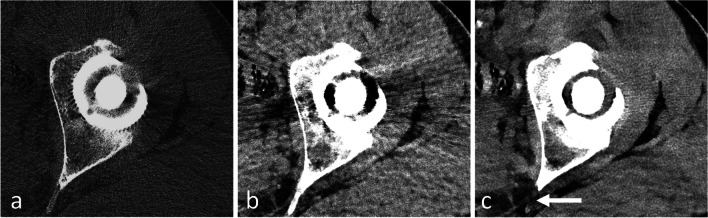
Metal artifact reduction: transverse reformats of PCD-CT of a left total hip replacement acquired at 140 kVp with tin filtration, and reconstructed using different parameters, which affect the metal artifacts and the assessment of the components. **a** Bone kernel, **b** virtual monoenergetic image (120 keV) with soft tissue kernel, and **c** virtual monoenergetic image (120 keV) with soft tissue kernel, and iterative metal artifact reduction algorithm (same window level as in** b**). Note that the assessment of soft tissue is best performed with the iterative metal artifact reduction algorithm (**c**), but these algorithms create artifacts mimicking areas of osteolysis (arrow). Bone structures are therefore best assessed without such algorithms (**a**)

The main advantage of PCDs is their ability to discriminate the energy of photons with an excellent energy resolution enabling the differentiation of materials that have similar attenuation coefficients but different atomic composition. This further enhances the accuracy of material classification and provides more comprehensive information about tissue composition (Figs. 4 and 5). A material decomposition algorithm requires at least two energy levels to separate a tissue into a combination of two known materials. Unlike DECT, PCD-CT offers the possibility to extract information on more than two energy levels, thereby enhancing tissue characterization and potentially leading to increased accuracy. A clear example of the potential of PCD-CT systems is the discrimination between calcium pyrophosphate, monosodium urate, or hydroxyapatite crystals for the diagnosis of the various crystal arthropathies [28] (Figs. 4 and 6). The use of the X-ray spectral information also allows for the reduction of beam-hardening and metal artifacts commonly present in CT imaging, without increasing radiation exposure (Fig. 7).

#### Current technical limitations: energy resolution, charge sharing, pile-up effect

Besides the remarkable advantages offered by PCD-CT compared to conventional CT, challenges persist [15]. Although much improved compared to EIDs, there are still limitations to the energy resolution capabilities of PCDs, which can affect material differentiation, particularly when resolving very close energy levels. Moreover, issues like charge sharing and pile-up can introduce inaccuracies in data interpretation (Fig. 1b). These phenomena pose significant challenges in accurately measuring particle interactions and handling rapid event rates in PCD-CT systems. Charge sharing is characterized by signal diffusion across adjacent detector elements. Individual events are then registered as multiple simultaneous interactions at lower energy degrading both the spatial and energy resolutions, thus reducing material decomposition capabilities and image contrast-to-noise ratio [29–31]. Similarly, pile-up, where multiple events are mistakenly registered as one due to their close temporal proximity, distorts the measured energy of individual events, impacting the accuracy of energy assessment and material differentiation in the system. As a result, overall image quality can be affected. For instance, these effects can lead to CT number inaccuracies, particularly for low- and high-attenuating materials like air and bone [32, 33]. However, effective correction methods have been developed and integrated into PCD-CT systems to mitigate or minimize the impacts of pulse pile-up and charge sharing, thereby preserving overall image quality and accuracy [34–36]. Ongoing research aims to refine PCD-CT, addressing these limitations and unlocking its full potential for enhanced clinical imaging and diagnosis.

### Current and future clinical applications of PCD-CT in MSK imaging

In this section, we provide an overview of the potential applications of PCD-CT based on its three main technical characteristics: high spatial resolution, low noise and decreased radiation exposure, and spectral resolution (Table 1). It should be noted that the advantages of PCD-CT with regard to these technical features could be used in combination (e.g., low noise at lower dose) but one advantage can be used to its full potential (highest obtainable resolution at full dose) if of clinical interest. The scientific evidence in this field is only emerging.

**Table 1:** Potential clinical applications of PCD-CT based on its technical features

	Spectral capabilities *- Material classification* *- Metal artifact reduction*	Spatial resolution - *High-resolution CT*	Radiation dose *- Low dose CT*
Trauma	Detection of bone marrow edema	Cortical and trabecular fracture detection	4D CT for chronic joint instability
Osteoporosis		Qualitative and quantitative bone architecture	
Osteoarthritis	Multicontrast compositional imaging of joint tissues	Qualitative and quantitative bone architecture	
Crystal arthropathies	Differentiation between different types of crystal deposits	Detection and quantification of crystal deposits	
Oncology		Detection and characterization of bone lesions	Low-dose whole body CT for the detection of skeletal metastases (multiple myeloma, solid cancers)
Pre-operative imaging	CT arthrography and virtual non-contrast CT: assessment of loose bodies and preoperative planning based on 3D reconstructions		Low-dose pre-operative assessment of bone and joint morphology
Post-operative imaging	Monoenergetic images of orthopedic hardware and periprosthetic complications		

#### High-resolution imaging

**Fig. 8:**
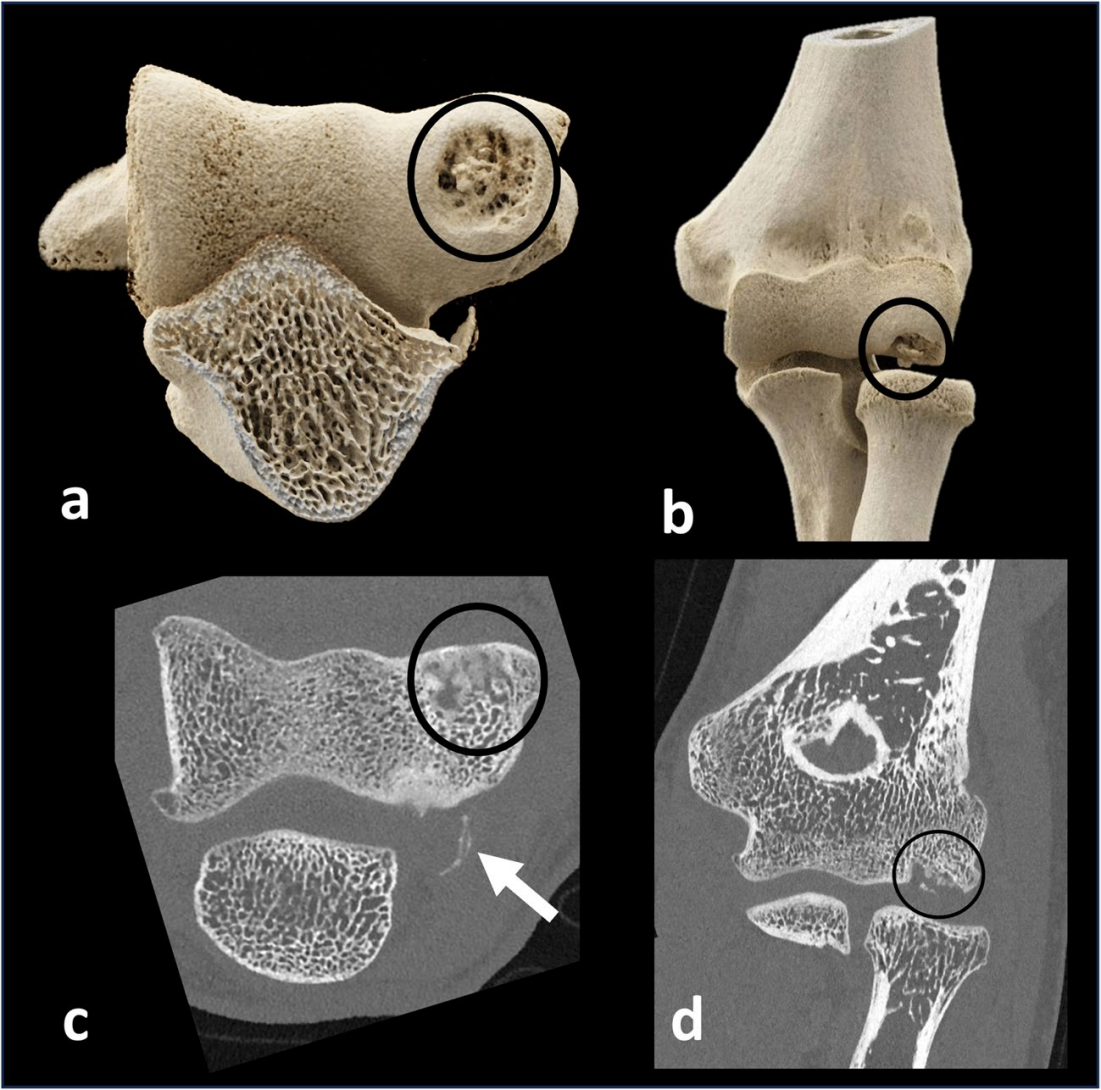
Improved confidence for the classification of osteochondritis dissecans in the elbow. Cinematic rendering technique (**a**, **b**) of an osteochondritis dissecans lesion of the elbow and greyscale images (**c**, **d**) providing high-resolution assessment on the size, and location of the osteochondral lesion (circle), as well as of the mineralized loose body (arrows)

The superior spatial resolution provided by PCD-CT is particularly useful in musculoskeletal imaging, notably for the imaging of bone and mineralized structures [37–40]. Higher spatial resolution compared to EID-CT provides better visualization of the fine details of cortical and trabecular bone and, therefore, improved detection of subtle cortical bone fractures, or the formation of callus (Fig. 3) [37, 38, 41, 42]. Thanks to this enhanced spatial resolution, PCD-CT might also offer an opportunity to assess bone architecture on clinical scanners [43]. The current clinical reference standard for the assessment of bone parameters is high-resolution peripheral quantitative CT (HR-pQCT) [44]. However, this modality is limited to the extremities. PCD-CT could potentially be applied to the axial skeleton to extract quantitative parameters of bone microarchitecture, such as cortical and trabecular thickness, separation, and number, in addition to bone mineral density. This would be valuable in assessing osteoporosis or other metabolic disorders of the bone. In theory, the high resolution (0.2 mm) might also allow for an increased diagnostic accuracy in characterizing bone tumors by enhanced visualization of matrix patterns, bone destruction, and periosteal reaction, although this must be confirmed in future studies. The higher resolution could also enhance the detection of subchondral (i.e., erosions, geodes, hyperostosis) and marginal (i.e., erosions, osteophytes) bone for the assessment of arthritis. Even though EID-CT has already been proven to offer complementary information to MRI for diagnosing and staging OCD, PCD-CT seems to be able to deliver an improved detection of mineralized loose bodies, due to the improved resolution (Fig. 8). This provides information whether a bone fragment is detached or still has an osseous bridge with enhanced certainty [45]. In addition, PCD-CT can provide spectral information, e.g., bone marrow edema mapping, or be combined to an arthrographic procedure to assess the interface between the lesion and surrounding bone. Altogether, this is expected to increase the diagnostic confidence in staging the OCD lesion. For all these applications, especially those concerning children and young patients, clinical studies should determine the optimal imaging strategies to comply with the ALARA (as low as reasonably achievable) principle.

#### Decreased noise and decreased radiation dose

The higher dose efficiency in PCDs may lead to improved image quality at the same dose. However, most musculoskeletal applications focus on high-contrast objects (i.e., imaging of mineralized tissue), and consequently higher noise levels can be tolerated without affecting performance [19, 24]. Therefore, it is more interesting to use the higher dose efficiency of PCDs to reduce the radiation dose while maintaining the diagnostic performance. For instance, an experimental PCD-CT system demonstrated lower image noise and improved reproducibility in assessing bone microstructure at a dose similar to EID-CT [46]. But on the other hand, a dose reduction by a factor of around 2 could be obtained without impairing performance in the evaluation of bone mineral density and bone microstructure [46].

Other initial studies and small case series have shown promise for dose reductions ranging from around 20% up to as high as 80% compared with EID-CT, while improving the visibility and sharpness of bone microarchitecture and disease-related lytic bone lesions and other abnormalities, as well as overall image quality [39, 47–50]. The level of dose reduction in the pediatric population is similar or even greater than that in the adult population, but this also depends on the willingness of radiologists to accept higher noise levels particularly in pediatric (CT) imaging.

While dose reductions can be applied to all clinical scenarios in musculoskeletal imaging, it is particularly useful for whole-body CT in the initial workup or serial imaging of patients with multiple myeloma or bone metastases, or in applications in younger patients, especially where radiosensitive organs are included in the scanned area [51–53]. For instance, reduced-dose PCD-CT has been used to mitigate the dose in hip and shoulder CT scans prior to joint replacement surgery, preoperative pelvic CT in younger patients with femoroacetabular impingement [52], and pre- or postoperative spinal CT [54, 55]. In theory, the spectral imaging potential of PCD-CT could allow the assessment of bone marrow, for which MRI remains the reference. The performance of PCD-CT in detecting and classifying bone marrow lesions will have to be investigated in future studies.

#### Spectral resolution: material classification and metal artifact reduction

Over the past decades, an increasing number of musculoskeletal applications of spectral imaging have been developed using DECT, of which the most common include the characterization and quantification of crystal deposits in patients with crystal arthropathies (gout and calcium pyrophosphate deposition disease), metal artifact reduction in postoperative imaging using virtual monoenergetic images, and the detection of bone marrow lesions in trauma patients thanks to virtual non-calcium images [19]. These applications generally rely on extracting the spectral information using various image reconstruction methods such material quantification, virtual non-contrast or virtual non-calcium images, and virtual monoenergetic images.

All these applications may be transferred to PCD-CT. In terms of spectral imaging capabilities, PCD-CT technology has the potential to provide diagnostic performance at least comparable to DECT, with higher spatial resolution and more efficient use of radiation dose, thereby overcoming the trade-off between spatial and spectral (multi-energy) resolutions (Figs. 4–6) [56]. For future work, artificial intelligence might be of assistance, not only for image reconstruction and segmentation algorithms generalizable to all CT systems, but also for enhanced spectral analysis and material classification.

##### Imaging of crystal arthropathies

One of the first applications of material classification in DECT and which can be transferred to PCD-CT is the assessment of crystal arthropathy, for the characterization and quantification of crystal deposits.

Spectral CT imaging has indeed found its way into clinical practice in crystal arthropathies, as demonstrated in international guidelines (e.g., EULAR [57]). DECT not only provides a diagnostic performance at least as accurate as ultrasound, but is also more reliable in identifying crystal deposits [58]. DECT further has the advantage of being quantitative, enabling the response to urate-lowering therapy to be monitored [59]. Moreover, DECT has become a key element in the classification criteria (e.g., ACR/EULAR) for gout and CPPD, for establishing the diagnosis in clinical research [60, 61].

However, the sensitivity of DECT remains to be improved, particularly in the early stages of crystal arthritides when crystal deposits are small and lowly concentrated [62].

In theory, PCD-CT has the potential to improve material classification (up to a factor of 2 in comparison to EID-based DECT, according to some CT manufacturers). However, these claims have yet to be demonstrated in clinical practice. To the best of our knowledge (and at the time of writing), the only studies investigating the impact of PCD-CT in crystal arthropathies were performed using a preclinical PCD-CT scanner (MARS Bioimaging®) with synthetic crystal phantoms and ex vivo human samples (Figs. 4 and 6) [28, 63, 64].

Theoretically, owing to its technical features, including but are not limited to its spectral capabilities, PCD-CT could contribute not only to the diagnosis and therapy response monitoring in gout, but also to a better understanding of the disease pathophysiology and the role played by the different crystal types, the differentiation between the types of calcium crystals (CPP and BCP) being limited with current DECT technology (Fig. 6) [28, 65, 66].

First, with its improved spatial resolution, PCD-CT could contribute to earlier diagnosis by improving the detection of tiny crystal deposits (single monosodium urate (MSU) and calcium pyrophosphate (CPP) crystals being each smaller than 0.02 mm in length), which is currently one of the main limitations of DECT in the early stages of gout and CPPD disease [62, 67]. An ongoing phantom study has shown that a point-of-care PCD-CT system (MARS Bioimaging) can identify twice as small MSU and CPP crystal deposits with comparable accuracy to DECT [68]. The minimum pixel sample size required to distinguish between MSU and CPP within any crystal aggregate/lesion decreased from 3 pixels (0.4-mm diameter) with DECT to 2 pixels (0.2-mm diameter) with PCD-CT. This finding is supported by proof-of-concept studies in which this PCD-CT system outperformed other clinical imaging techniques (including DECT) for the identification of MSU and CPP crystals in a gouty finger with subcutaneous tophus and in menisci and hyaline cartilage harvested from osteoarthritic knees, approaching the high spatial resolution of ultrasound while providing superior crystal characterization capability (Fig. 4) [28, 64].

Second, with its superior multi-energy, quantitative imaging capabilities, and reduced electronic noise, PCD-CT has the potential to improve the characterization of calcium crystal aggregates (i.e., CPP vs. basic calcium phosphate (BCP) similar to the classification of calcium-containing urinary stones [69]). The identification of MSU deposits within more challenging backgrounds such as hyaline cartilage or fibrocartilage in joints or the spine could also be improved. Previous phantom studies, supported by ex vivo analyses, have shown promising results in slightly improving the diagnostic performance for such a clinical task compared with DECT [28, 63, 64]. However, crystal classification with current PCD-CT technology remains far from perfect, and further developments (optimization of image acquisition and reconstruction protocols, including radiomics analysis) are needed.

Finally, PCD-CT has also the potential to enable a more accurate and reliable quantification of MSU and calcium crystal deposits (CPP and BCP), in analogy with recent results of phantom studies on the accuracy of CT numbers and bone mineral density measurements [43, 70]. These volume measurements are increasingly used in monitoring the response to urate-lowering therapy in patients with gout [71], as well as in gout patient education.

##### Imaging of metallic hardware

Since the early days of CT, imaging of metallic objects such as orthopedic hardware and joint prostheses has been quite challenging due to the substantial image artifacts. Common clinical indications in this context include periprosthetic infection or fractures, and hardware complications such as hardware fracture or loosening. Metal artifacts are caused by factors such as photon starvation, beam hardening, scatter, and electronic noise. As a result, the visualization of anatomical structures adjacent to metal hardware is compromised and may lead to decreased diagnostic confidence or even an impossibility to assess the area of interest.

The severity of metal artifacts is influenced by a number of factors. These include, on one hand, the composition, size, shape, and location of the metallic objects, and on the other hand, the CT acquisition parameters and reconstruction algorithms.

**Fig. 9:**
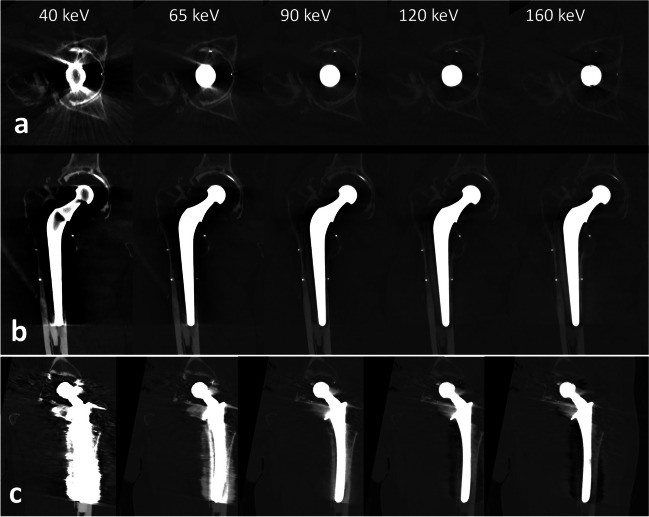
Total hip replacement imaging with PCD-CT virtual monoenergetic images: **a** transverse and (**b**) coronal reformats of a right total hip replacement and coronal reformats of a left total joint replacement (**c**), with increasing energy levels. The acquisition was performed at 140 kVp, with soft tissue kernel and no iterative metal artifact reduction. PCD-CT generates virtual monoenergetic images that range from 40 to 160 keV that progressively reduces metal artifacts around total hip replacements, allowing for improved evaluation of the metal-bone interface and surrounding structures. Note that in those clinical cases, the “sweet spot” of optimal metal artifact reduction is around 90 keV, with some degree of degradation in image quality at higher keV

**Fig. 10:**
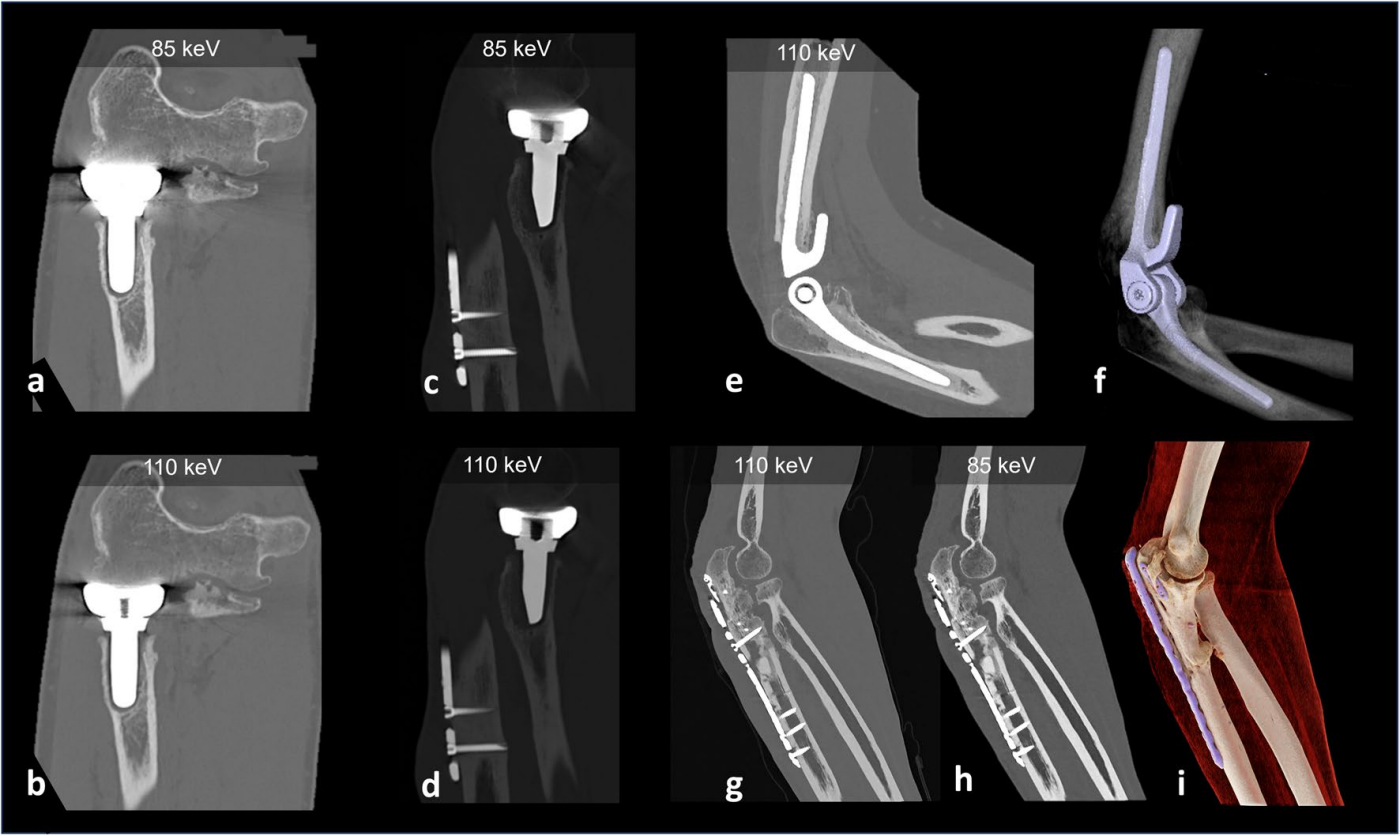
Metal artifact reduction in the elbow. PCD-CT of joint replacements (**a**,** b**,** e**,** f**) and metal hardware (**c**, **d**, **g**–**i**) of the elbow with acquisitions at 140 kVp, with tin filtration. Virtual monoenergetic images at different levels are shown. **f**, **i** Volume rendering images. As PCD-CT also comes by default with an increased number of bits for the DICOM images (16-bits), it provides more shades of gray/extended CT window scale. The images can be optimized with the combination of spectral shaping, high keV and a wide WW/WC of, e.g., 10.000/4000

Several methods have been used to mitigate these artifacts. During the acquisition, the adjustment parameters (e.g., high kilovoltage (kVp), slow rotation times, and low pitch) can help decrease metal artifacts at the expense of increasing the radiation dose. In the reconstruction domain, metal artifact reduction (MAR) techniques based on iterative reconstruction algorithms can be used and offer choices based on the metal type and location (e.g., hip or shoulder implants, pacemaker, or coils) [72]. However, these methods tend to degrade image sharpness due to limited kernel strength. They also may induce some artifacts mimicking osteolysis (Fig. 7). Current EID-based DECT scanners may reconstruct less artifact-prone, high keV images using the spectral information. However, these solutions require a tradeoff between the spatial resolution and the spectral information. With PCD-CT, the spectral information may be used to produce low artifact monoenergetic images without sacrificing spatial resolution. It was shown that hard reconstruction kernels and thin slices (0.2 mm) with PCD-CT offer sharper delineation of bone-implant interfaces compared to EID-CT [73]. In terms of acquisition parameters, the strategies used for reducing metal artifacts in EID-CT may be similarly applied to PCD-CT. High kVp allows for reduction of metal artifacts, especially when combined with additional tin filtration [74]. Despite the reduction in bone contrast when applying additional tin filtration, it seems the method of choice to reduce artifacts in total hip replacements (Figs. 7 and 9). The same applies to smaller metallic hardware, e.g., wrist prosthesis or screws, where additional tin filtration allows for reduction of metal artifacts (Fig. 10). A current downside of applying an extra tin filtration is that the user is not able to reconstruct the so-called spectral post-processing (SPP) files, limiting the ability to interactively change the keV settings. However, images may still be reconstructed between 60 and 190 keV. The reconstruction of monoenergetic images for implants is most effective within the 90–130 keV range, with the ideal keV level varying depending on the hardware (Fig. 9) [55, 75]. It is also to note that the combination of high keV images with MAR techniques effectively reduces metal artifacts in adjacent soft tissues, but it is less suitable for the visualization of the bone-implant interface due to limited image sharpness [76].

##### Other, emerging applications

In addition to the aforementioned applications, which are already incorporated into clinical practice at least in some institutions using DECT, the advent of PCD-CT may accelerate the emergence of new applications of spectral imaging, given ongoing advances in the research domain.

Potentially, PCD-CT could provide sufficient contrast resolution to improve the imaging of soft tissue lesions, in particular collagen-rich structures such as intervertebral discs or ligaments, as previously attempted using DECT [77–79]. Others have aimed to use the potential of PCD-CT to improve high-contrast resolution to image cartilage without contrast (as typically done in CT arthrography), by optimizing the level of monenergetic images in cadavers [80, 81].

Others have attempted a compositional analysis of cartilage, by assessing glycosaminoglycan (GAG) content and distribution throughout cartilage, similar to what is performed at MRI using dGEMRIC imaging [82, 83]. It was shown that anionic iodine or gadolinium-based contrast agents are distributed inversely to negatively charged GAGs throughout the cartilage, hereby providing an indirect quantification of the GAG content [84, 85]. Furthermore, by utilizing a multi-material decomposition technique, it was possible to distinguish the deep zone articular cartilage from underlying subchondral bone, which exhibit similar attenuation levels. Likewise, Paakkari et al. successfully employed an experimental PCD-CT setup to quantify a cationic iodinated CA4 + (proportionally distributed to GAGs) and a non-ionic gadolinium-based gadoteridol (reflecting water content) contrast agents within human osteochondral tissue samples [86].

Therefore, PCD-CT has the potential to provide a quantitative analysis of both the cartilage and the adjacent subchondral bone structure, hereby representing an interesting tool to further clarify the intricate role of these tissues in the pathogenesis of OA [87].

In terms of material classification, other potential applications of PCD-CT may extend previous work from DECT, including the characterization of hemosiderin deposits in giant cell tumors of the tendon sheath, as well as the detection of metal debris in metallosis [19].

PCD-CT has also the potential to improve the measurement of fat fraction in various tissues, as already shown in the liver [88]. If their accuracy is confirmed for the bone or muscle, fat fraction measurements available on clinical scanners, including in a retrospective manner, could be used as an opportunistic surrogate marker to predict certain morbidities or provide insight into the pathophysiology of various endocrine, metabolic, or hematological disorders [89–91].

In CT arthrography, the spectral capabilities of PCD-CT may be used to obtain virtual non-contrast images to detect intra-articular loose bodies, or provide 3D reconstructions for preoperative planning without the need to perform additional scans, limiting patient radiation exposure [19, 92].

Finally, the higher dose efficiency of PCD-CT systems could further promote emerging applications of 4D CT, which has already been successfully used for the imaging of wrist or subtalar instability on EID-CT systems [93, 94].

At this stage, further preclinical and clinical studies are required to validate the performance of PCD-CT for all these potential applications.

### Financial considerations

The installation and maintenance of PCD-CT systems are typically associated with higher costs compared to conventional CT systems. For example, at the time of writing (end of 2023), the cost of the presently FDA-approved PCD-CT, which is a dual-source, dual-photon-counting detector system, is approximately two to three times higher than that of a standard CT system. Technical constraints for the electrical system also contribute to higher installation costs and maintenance costs are typically higher. Eventually, the added-value of PCD-CT systems would require a cost-effectiveness analysis that considers the full range of clinical applications of the scanner, not limited to musculoskeletal applications.

## Conclusion

In summary, PCD-CT has interesting theoretical advantages over EID-CT, all of which can be useful in musculoskeletal imaging. These advantages include higher spatial resolution, lower noise and radiation dose, as well as intrinsic spectral imaging capabilities. Therefore, PCD-CT has the potential to enhance CT imaging across its existing range of applications, particularly the improved visualization of bone structures, reduced radiation exposure, and promising prospects in addressing crystal arthropathies and imaging metallic hardware. Additionally, technological advancements may pave the way for the emergence of new applications, such as optimized soft tissue contrast, or multiple contrast imaging for compositional imaging of tissues. Nevertheless, the theoretical enhancements in diagnostic performance still need validation on a larger clinical scale, the current evidence mainly consisting in experimental feasibility studies. For each application, the diagnostic performance must be rigorously evaluated clinically, and the potential limitations, image artifacts, or pitfalls described thoroughly. While most novel applications will rely heavily on post processing, standardization of these tools across platforms will be required for wide clinical adoption.

Additionally, technological challenges remain to be addressed to further improve PCD-CT, including energy resolution, charge sharing, and pile-up. From an economical perspective, the cost of PCD-CT is currently much higher than conventional EID-based systems. Consequently, the cost-effectiveness of these systems should be thoroughly assessed. The near future will determine whether PCD-CT represents a true revolutionary change in CT imaging, or merely a significant leap forward.

## Abbreviations

DECT: Dual energy computed tomography
CT: Computed tomography
EID: Energy-integrating detectors
PCD: Photon-counting detectors
